# PIKfyve Deficiency Exacerbates Radiation-Induced Intestinal Toxicity

**DOI:** 10.3390/toxics14050434

**Published:** 2026-05-14

**Authors:** Aoqiang Ji, Xing Shen, Chunan Zhao, Zhaopeng Weng, Xuewen Zhang, Kai-Kai Yu, Shuang Xing, Xinlong Yan, Zuyin Yu

**Affiliations:** 1 Academy of Military Medical Sciences, Academy of Military Science, Beijing 100850, China; aoqiang_ji@163.com (A.J.); shen_xingjyk@163.com (X.S.); chunanzhao117@outlook.com (C.Z.); wzp_anl@163.com (Z.W.); zhangxw94@163.com (X.Z.); yukaikai321@163.com (K.-K.Y.); xshuang6564@163.com (S.X.); 2 Beijing Key Laboratory of Environmental and Viral Oncology, College of Chemistry and Life Science, Beijing University of Technology, Beijing 100124, China; 3 School of Life Science, Anhui Medical University, Hefei 230032, China

**Keywords:** radiation toxicology, intestinal acute radiation syndrome, PIKfyve, epithelial barrier, radiation countermeasures, translational radiobiology

## Abstract

Background: Intestinal acute radiation syndrome (IARS) represents a life-threatening component of acute radiation syndrome with limited effective countermeasures. Understanding molecular determinants governing intestinal epithelial resilience to ionizing radiation is critical for developing radiation toxicity mitigation strategies. Objectives: This study investigates the role of PIKfyve, a phosphoinositide kinase essential for endolysosomal homeostasis, in modulating radiation-induced intestinal toxicity. Methods: We utilized an inducible intestinal epithelial-specific PIKfyve-knockout mouse model (PIKfyve cKO) subjected to 10 Gy abdominal irradiation. Intestinal toxicity was assessed through histopathology, barrier permeability (FD4 assay), apoptosis markers, and transcriptomic profiling. Small intestinal organoids were employed for mechanistic validation. Results: PIKfyve deletion alone did not perturb normal gut architecture but precipitated severe post-irradiation toxicity, including villous atrophy, crypt hypoplasia, and massive crypt-cell apoptosis. Barrier dysfunction was evidenced by elevated serum FD4 and heightened systemic pro-inflammatory cytokines, culminating in markedly increased mortality. Transcriptomic analysis revealed potentiated DNA-damage signaling and amplified inflammatory cascades in PIKfyve-deficient intestines. Conclusions: These findings identify PIKfyve as a critical guardian of intestinal epithelial integrity against radiation toxicity. Given emerging PIKfyve inhibitors in cancer therapy, our results raise important safety considerations for clinical radiotherapy and position PIKfyve as a potential target for radiation toxicity mitigation.

## 1. Introduction

Ionizing radiation is widely utilized in cancer radiotherapy, nuclear medicine, and industrial applications, yet radiation-induced toxicity remains a significant dose-limiting factor compromising treatment efficacy and patient quality of life [1]. The gastrointestinal tract represents one of the most radiosensitive organs, with acute radiation-induced intestinal injury developing within days of exposure, clinically manifesting as severe diarrhea, abdominal pain, and hematochezia [2,3]. Beyond therapeutic contexts, accidental radiation exposure scenarios—such as nuclear accidents or radiological terrorism—necessitate effective medical countermeasures against intestinal acute radiation syndrome (IARS), for which no FDA-approved prophylaxis currently exists [4,5]. This critical unmet need underscores the importance of identifying molecular targets that govern intestinal epithelial resilience to radiation toxicity.

The pathophysiology of radiation-induced intestinal toxicity involves a complex cascade initiated by direct DNA damage to crypt stem cells, subsequent impairment of epithelial renewal, and loss of mucosal barrier integrity [6]. Once the epithelial barrier is breached, luminal bacterial translocation triggers robust inflammatory responses that amplify tissue damage, creating a vicious cycle of injury. Intestinal epithelial cells, characterized by lifelong rapid self-renewal, not only mediate nutrient absorption but also constitute the critical barrier preventing microbial invasion and orchestrating mucosal immunity [7,8]. Understanding the molecular determinants that preserve epithelial integrity under radiation stress is therefore essential for developing targeted toxicity mitigation strategies.

PIKfyve (phosphoinositide kinase, FYVE-type zinc finger containing) is the sole known kinase catalyzing the phosphorylation of phosphatidylinositol-3-phosphate (PI3P) to phosphatidylinositol-3,5-bisphosphate [PI(3,5)P2], a lipid second messenger critical for endolysosomal homeostasis, vesicular trafficking, and autophagy regulation [9,10,11,12]. Pharmacological inhibition of PIKfyve has emerged as a promising therapeutic strategy in cancer treatment, with inhibitors such as apilimod demonstrating efficacy in B-cell lymphoma and solid tumors by disrupting lysosomal function and autophagy [13,14,15]. However, the potential impact of PIKfyve modulation on normal tissue radiation toxicity remains completely unexplored—a critical knowledge gap given the concurrent use of radiotherapy and emerging PIKfyve-targeted therapies.

Given PIKfyve’s essential role in maintaining intestinal epithelial homeostasis and its emerging significance in cancer therapeutics, we hypothesized that PIKfyve deficiency would exacerbate radiation-induced intestinal toxicity. To test this hypothesis, we generated an inducible intestinal epithelial-specific PIKfyve-knockout mouse model and subjected these mice to clinically relevant abdominal irradiation. Our findings reveal that PIKfyve serves as a previously unrecognized protective factor against radiation-induced intestinal toxicity, with important implications for radiation countermeasure development and clinical safety assessment of PIKfyve inhibitors in radiotherapy patients.

## 2. Materials and Methods

### 2.1. Experimental Animals

The PIKfyve intestinal epithelial gene knockout mice were obtained from The Jackson Laboratory16 and subsequently propagated through independent breeding. After breeding and cultivation, mice with the desired genotype were obtained. The induction and irradiation experiments were conducted when the male mice reached an appropriate weight (22–26 g) and were 6–8 weeks old. Animals were housed in SPF barrier facilities (22 ± 2 °C, 50–60% humidity, 12 h light/dark cycle) with ad libitum access to standard chow and water.

### 2.2. Experimental Groups

Mice were randomly assigned to the following groups: (1) PIKfyve^fl/fl^ (Cre-negative) mice receiving vehicle control; (2) PIKfyve^fl/fl^;Villin-Cre/ER^T2^ (cKO) mice receiving vehicle control; (3) PIKfyve^fl/fl^ mice receiving tamoxifen-only (tamoxifen control); (4) PIKfyve^fl/fl^;Villin-Cre/ER^T2^ mice receiving tamoxifen, with or without 10 Gy (or 13 Gy for survival studies) abdominal irradiation. A detailed summary of the experimental design is provided in Table 1.

**Table 1:** Summary of experimental design and group allocation for all figures.

Figure Number	Experiment	Group Design	Mice per Group	Groups	Mice
**Figure 1A–C**	Histology/BrdU (3.5 d)	WT; cKO	3	2	6
**Figure 2A**	Histology/BrdU (3.5 d)	IR WT; IR cKO	3	2	6
**Figure 2D**	Olfm4 IHC staining	WT; cKO; IR WT; IR cKO	3	4	12
**Figure 2F**	Survival (13 Gy)	IR WT; IR cKO	10	2	20
**Figure 3A**	FITC-Dextran permeability (10 Gy)	WT; cKO; IR WT; IR cKO	6	4	24
**Figure 3B**	Claudin1 IF staining	WT; cKO; IR WT (6 h); IR cKO (6 h); IR WT (24 h); IR cKO (24 h); IR WT (3.5 d); IR cKO (3.5 d)	3	8	24
**Figure 3C**	RT-PCR	WT; cKO	3	2	6
**Figure 3D**	RT-PCR	IR WT; IR cKO	3	2	6
**Figure 3E**	Caspase-3 IHC	WT; cKO; IR WT; IR cKO (6 h)	3	4	12
**Figure 3G**	TUNEL	WT; cKO; IR WT; IR cKO (6 h)	3	4	12
**Figure 4A**	Intestine organoid	WT; cKO Intestinal crypts were isolated from mouse small intestine for organoid culture and passaging. Subsequent experiments involved direct irradiation of the organoids without the use of mice.	3	2	6
**Figure 4H**	Alox12b IF staining	WT; cKO; IR WT (6 h); IR cKO (6 h); IR WT (24 h); IR Cko (24 h) The mice employed for this experiment were those from which specimens were collected for Figure 3B.	/	/	/
**Total**					134

**Figure 1:**
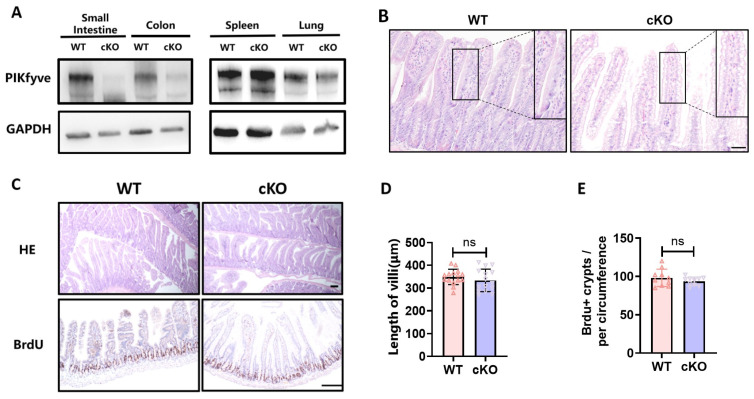
After knocking out PIKfyve in intestinal epithelial cells, there was no significant effect on the length of intestinal villi and the number of proliferative crypts. Mice were continuously induced with tamoxifen for 5 days. On the 7th day after completion of induction, samples were collected. (**A**) WB Results Validate Specific Knockout of PIKfyve in the Intestine. (**B**) HE Staining Results Show Significant Vacuolization in Intestinal Villous Epithelium After PIKfyve Knockout. (**C**) The “Swiss roll” sections were utilized to display the length of villi, while BrdU immunohistochemistry staining was employed to demonstrate the number of proliferative crypts. (**D**) Statistical Analysis of Villus Length in Each Group. (**E**) The statistical results of BrdU+ crypts in each intestinal loop. ns (not significant), *p* > 0.05, *n* = 3, Scale bar, 100 μm. Original WB date, see Appendix A.

**Figure 2:**
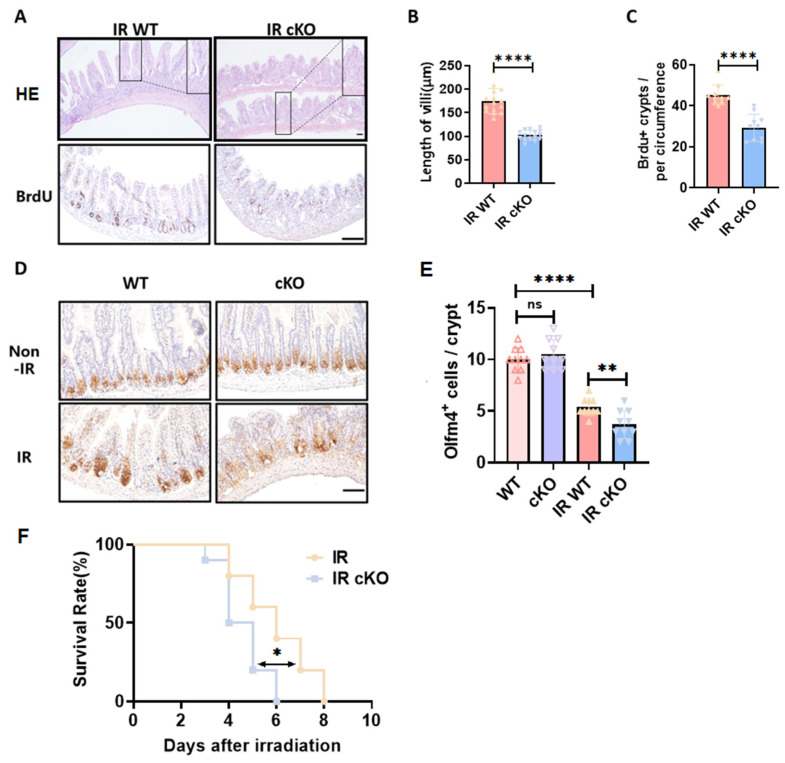
Deletion of PIKfyve in intestinal epithelial cells results in structural damage to the intestines and reduces the expression of stem cells within the crypts post-irradiation. (**A**) The “Swiss roll” sections of the intestine stained with hematoxylin and eosin (HE) and immunohistochemistry for BrdU staining of crypts. (**B**) Statistical Analysis of Villus Length. (**C**) Statistical Results for BrdU+ Crypt Count in Each Intestinal Ring. (**D**) Immunohistochemistry Staining Results for Olfm4 in Intestinal Crypts. (**E**) Statistical Results for Olfm4+ Cells Count in Each Crypt. *n* = 3. (**F**) The survival status of mice following 13 Gy irradiation (*n* = 10); the 13 Gy dose was selected to achieve measurable mortality differences within the 14-day observation window, as 10 Gy produced insufficient mortality in wild-type controls. * *p* < 0.05, ** *p* < 0.01, **** *p* < 0.0001 vs. wild type (WT), ns, not significant, *n* = 3, Scale bar, 100 μm.

**Figure 3:**
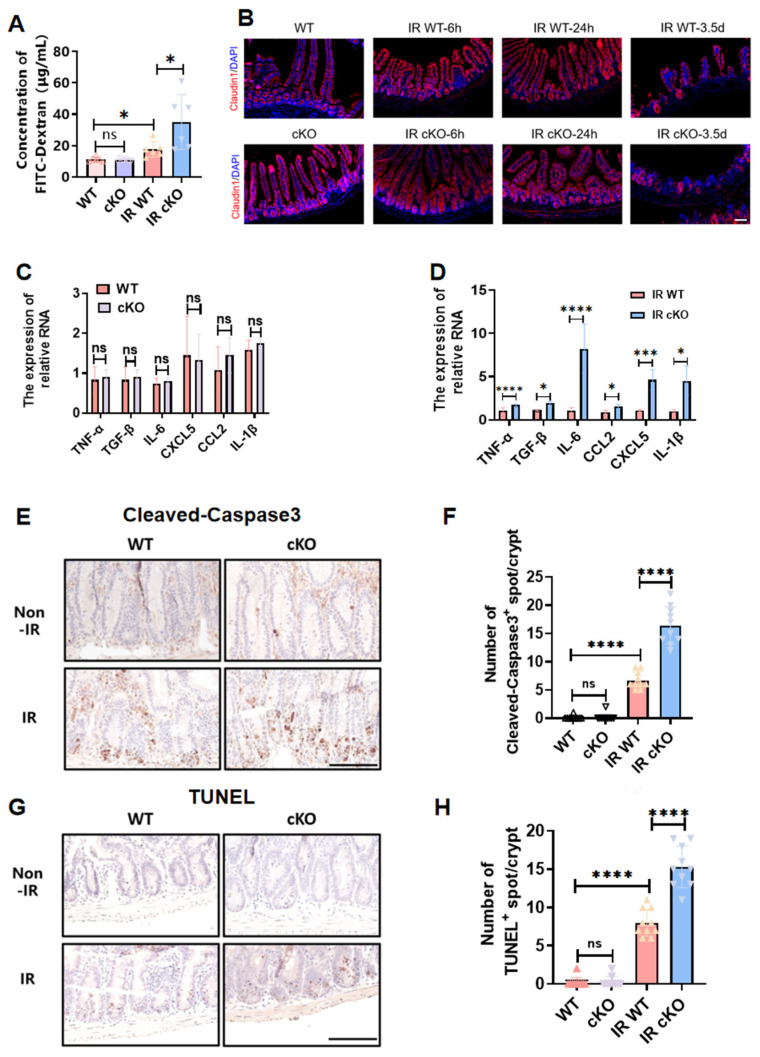
Intestinal epithelial PIKfyve deletion increases intestinal permeability post-irradiation, exacerbates the inflammatory response, and enhances crypt cell apoptosis. (**A**) Statistical Results of FD4 Content in Peripheral Blood Serum of Mice in Each Group, *n* = 6. (**B**) Representative images showing Claudin-1 expression in small intestinal epithelial cells. (**C**) Expression Levels of Inflammatory Factors in the Non-Irradiated Group, *n* = 3. (**D**) Expression Levels of Inflammatory Factors in Both Groups of Mice Post-Irradiation, *n* = 3. (**E**) Immunohistochemistry Staining Results for Cleaved-Caspase 3 in Intestinal Crypts. (**F**) Statistical Results for Cleaved-Caspase 3^+^ Counts in Each Crypt. (**G**) TUNEL Staining Results for Intestinal Crypts. (**H**) Statistical Results for TUNEL^+^ Counts in Each Crypt. ns (not significant) *p* > 0.05, * *p* < 0.05, *** *p* < 0.001, **** *p* < 0.0001 vs. wild type (WT), *n* = 3, Scale bar, 100 μm.

**Figure 4:**
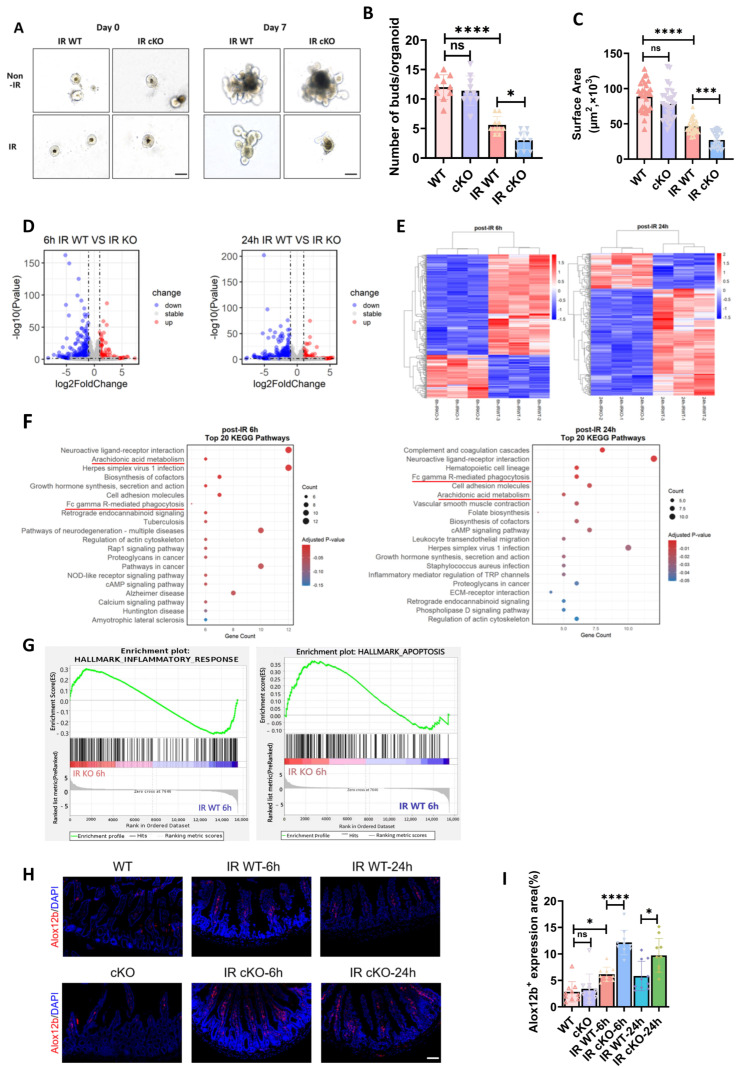
Intestinal epithelial deletion of PIKfyve suppresses post-irradiation organoid growth, triggers inflammation, and promotes apoptosis. (**A**) Representative morphological image of mouse small-intestinal organoids at day 0 (**left** panels, showing both non-irradiated and irradiated wild-type organoids at similar baseline morphology) and day 7 post-irradiation (**right** panels). (**B**) Quantification of budding efficiency in mouse small-intestinal organoids. (**C**) Quantitative analysis of growth area in mouse small-intestinal organoids. (**D**) Volcano plots of differentially expressed genes in intestinal organoids from PIKfyve-knockout versus control mice at 6 h and 24 h post-irradiation. (**E**) Heat maps of differentially expressed genes in intestinal organoids from PIKfyve-knockout and control mice at 6 h and 24 h post-irradiation. (**F**) Top 20 KEGG pathway enrichment analyses of intestinal organoids from PIKfyve-knockout and control mice at 6 h and 24 h post-irradiation. (**G**) GSEA enrichment of inflammation- and apoptosis-related genes at 6 h post-irradiation. (**H**) Representative images showing Alox12b expression in small intestinal epithelial cells. (**I**) Statistical Results for Alox12b^+^ area in Each intestine. ns (not significant) *p* > 0.05, * *p* < 0.05, *** *p* < 0.001, **** *p* < 0.0001 vs. wild type (WT), *n* = 3, Scale bar, 100 μm.

### 2.3. Mouse Irradiation and Treatment

The biological irradiator SHARP 100(Raycision Medical Technology Co., Ltd., Hefei, China) was used for local irradiation. The settings were as follows: voltage 220 kV, current 25 mA, dose rate 1.325 Gy/min, and irradiation dose 10 Gy (for histopathology, barrier function, and molecular analyses) or 13 Gy (for survival studies, selected to achieve measurable mortality differences within the observation window while remaining sub-lethal for wild-type mice). Prior to irradiation, mice were anesthetized with Tribromoethanol (Abmole, Shanghai, China, M14641) injected into the abdominal cavity. Subsequently, mice were immobilized in a custom-made mouse irradiation box, and the chest and lower abdominal regions were shielded with lead plates, exposing only the entire abdominal area. Mice received tamoxifen (Shanghai Aladdin Biochemical Technology Co., Ltd., Shanghai, China, T137974) administration for 5 consecutive days (100 mg/kg, intraperitoneal injection, dissolved in corn oil) prior to irradiation; the same tamoxifen regimen was administered to all groups, including Cre-negative and vehicle controls, to control for potential tamoxifen-induced effects.

### 2.4. Western Blot Analysis

On the 7th day post-induction, tissues from the small intestine, colon, spleen, and lung of each group of mice were collected for protein extraction. After electrophoresis, proteins were transferred to a nitrocellulose (NC) membrane (Merck Millipore, Darmstadt, Germany, IPVH00010). The NC membrane was blocked with 5% skimmed milk (EpiZyme, Shanghai, China, PS112L)-TBST blocking solution for 2 h, followed by overnight incubation with primary antibodies (anti-PIKfyve, Abclonal, Wuhan, China, A6689; anti-GAPDH, Proteintech, Wuhan, China, 60004-1-Ig) at 4 °C. After washing off excess primary antibodies with TBST, the NC membrane was incubated with secondary antibodies (EpiZyme, Shanghai, China, LF102) for 1 h. After another round of TBST washing to remove excess secondary antibodies, the membrane was subjected to visualization and imaging using an enhanced chemiluminescence (ECL) detection system (Vazyme, Nanjing, China, E423-02).

### 2.5. Tissue HE Staining

At day 7 post-induction, mouse intestinal segments were collected, and “Swiss rolls” were prepared. Tissues were fixed in 4% paraformaldehyde (Servicebio, Wuhan, China, G1101-500ML) for 24 h. Subsequently, the intestinal tissues were dehydrated, embedded in paraffin, and sectioned along the longitudinal and transverse sections of the intestinal ring and “Swiss rolls” at a thickness of 5 μm. After deparaffinization, HE staining was performed, and the slides were observed under a microscope.

### 2.6. Tissue Immunohistochemical Staining

Tissue sections of mouse intestines were processed similarly to Section 2.5. Tissue sections were placed in citrate sodium repair solution (ZSGB-BIO, Beijing, China, ZLI-9065), underwent high-temperature repair for 15 min, and naturally cooled to room temperature. After blocking endogenous peroxidase activity (ZSGB-BIO, Beijing, China, ZLI-9311D) for 30 min at room temperature, tissues were incubated in 10% normal goat serum (ZSGB-BIO, Beijing, China, ZLI-9056) to block non-specific binding for 1 h at room temperature. Primary antibodies were diluted according to the recommended concentration, incubated with tissues, and placed in a humid chamber at 4 °C overnight. After rewarming, excess primary antibodies were washed off with PBS, and tissues were incubated with enzyme-labeled goat anti-mouse/rabbit IgG (ZSGB-BIO, Beijing, China, PV-6000) at room temperature for 1 h. After washing with PBS, DAB chromogenic solution (ZSGB-BIO, Beijing, China, ZLI-9019) was applied, and after counterstaining, dehydration, sealing, and drying, the slides were observed under a microscope. The following primary antibodies were used: anti-BrdU (1:600, Servicebio, Wuhan, China, GB12051-100), anti-Olfm4 (1:600, Cell Signaling Technology, Beverly, MA, USA, 39141), and anti-Cleaved-Caspase3 (1:600, Cell Signaling Technology, Beverly, MA, USA, 9661).

### 2.7. Tissue Immunofluorescence Staining

Tissue sections were processed similarly to Section 2.5. Sections were cooled to room temperature, washed with PBS, and permeabilized with 0.2% Triton X-100 (Servicebio, Wuhan, China, GC204003-100ml) for 15 min. After rinsing with PBS, endogenous peroxidase activity was blocked for 30 min. Subsequent blocking and antibody incubation were performed as described in Section 2.6. For fluorescent detection, sections were stained using a TSA fluorescence kit (Panovue, Beijing, China, 10288100100). After rinsing with PBS, sections were mounted with DAPI-containing fluorescence mounting medium (Huilanbio, Shanghai, China, HL061). Finally, images were captured under a fluorescence microscope. The following primary antibodies were used: anti-Claudin-1 (1:600, Proteintech, Wuhan, China, 28674-1-AP) and anti-Alox12b (1:200, Abclonal, Wuhan, China, A14703).

### 2.8. TUNEL Assay

TUNEL staining was performed using the Boster TUNEL detection kit (Boster Biological Technology, Wuhan, China, #MK1014-100). Tissue samples were fixed in 4% paraformaldehyde for at least 4 h, embedded in paraffin, sectioned, and dewaxed and rehydrated. Sections were incubated with freshly diluted Proteinase K (1:200 in 0.01 M TBS) at 37 °C for 15 min, and washed three times with 0.01 M TBS for 2 min each. After washing, 20 μL of labeling buffer per section was applied to keep the sections moist. Meanwhile, the labeling solution was prepared by mixing 1 μL of terminal deoxynucleotidyl transferase (TdT) and 1 μL of BIO-d-UTP with 18 μL of labeling buffer. Excess liquid was drained from the sections, and 20 μL of labeling solution per section was applied. The sections were incubated in a humidified chamber at 37 °C for 2 h. After labeling, sections were washed with 0.01 M TBS for 2 min, three times. Blocking solution (50 μL/section) was added, and sections were incubated at room temperature for 30 min. The blocking solution was discarded without washing. SABC-AP was diluted 1:100 in antibody diluent (10 μL of SABC-AP in 1 mL of antibody diluent), and 50 μL per section was applied. Sections were incubated at 37 °C for 60 min, and washed four times with 0.01 M TBS (pH 7.5) for 5 min each. Finally, BCIP/NBT color development was performed: BCIP/NBT stock solution (×20) was diluted 1:20 in 0.01 M TBS (pH 9.0–9.5), mixed thoroughly, and applied to the sections. Color development was carried out in the dark for 20–30 min (the duration could be appropriately extended if no background appeared). The reaction was terminated by thorough washing with water.

### 2.9. FD4 Content Measurement

Mice were fasted and deprived of water for 3 h on day 3 post-irradiation. Each mouse was orally administered 150 μL of 4 kDa FITC-dextran (referred to as FD4, Merck Millipore, Darmstadt, Germany, FD4-1G) at a concentration of 80 mg/mL. After 3 h of administration, 100 μL of blood was taken from the tail vein and incubated at 37 °C for 1 h before transferring to 4 °C for 2 h. After centrifugation at room temperature for 10 min at 3000× *g*, 20 μL of the upper serum was taken and diluted with 80 μL of water. FD4 solution and diluted serum were added to a 96-well plate, and absorbance was measured. FD4 content in the serum was calculated to reflect intestinal permeability.

### 2.10. Real-Time Quantitative PCR

RNA samples were extracted using Trizol (CWBIO, Taizhou, China, CW0580S), and after measuring RNA concentration, cDNA was obtained by reverse transcription using the HiScript III All-in-one RT SuperMix Perfect (Vazyme, Nanjing, China, R333-01) for qPCR kit. Primers were designed using Primer Premier 5.0 software, and their sequences are provided below (Table 2). RT-QPCR was performed using SYBR Green Pro Taq HS pre-mixed qPCR reagent (Vazyme, Nanjing, China, Q712) and the Roche LightCycler^®^ 96 Instrument (Roche, Basel, Switzerland). The reaction conditions were as follows: pre-incubation at 95 °C for 30 s, two-step amplification at 95 °C for 10 s, 60 °C for 30 s, and 45 cycles; dissolution at 95 °C for 10 s, 65 °C for 60 s, and 97 °C for 1 s. RT-QPCR results were analyzed using the 2-ΔΔCt method.

### 2.11. Mouse Small-Intestinal Organoid Experiment

Following the induction protocol, the entire small intestine was harvested from mice. The tissue was opened longitudinally, villi were removed, and the mucosa was washed fifteen times with sterile PBS. Crypts were isolated with EDTA (Beijing Solarbio Science & Technology Co., Ltd., Beijing, China, IE9040), resuspended in Matrigel (ABW, Shanghai, China, 082755), and seeded at 50 µL per well in 24-well plates. Upon Matrigel solidification, mouse small-intestinal organoid medium (ABW, Shanghai, China, MA-0817H006SP) was added. Cultures were monitored daily, passaged as required, and then irradiated. Post-irradiation growth was recorded, and RNA was extracted for sequencing.

Inclusion/exclusion criteria:

Mice were included only if they met the following pre-established criteria: (i) body weight within 22–26 g, (ii) normal baseline stool consistency, and (iii) completion of the full radiation protocol. Animals showing wound infection, anesthesia-related mortality within 24 h, or technical failure of irradiation were pre-specified for exclusion. No animals or data points were excluded from the final analyses.

Randomization and blinding:

Group allocation was randomized by a computer-generated random sequence stratified by body weight. Cage position on the rack and order of procedures were rotated daily to minimize environmental confounders. Cage location and order of procedures were randomized across groups to eliminate spatial and temporal biases. Investigators performing histological scoring, organoid imaging, and survival checks were blinded to treatment allocation; blinding was broken only after final data lock.

### 2.12. RNA Sequencing

Total RNA was extracted from freshly isolated organoids using FastPure Cell/Tissue Total RNA Isolation Kit V2 (Vazyme, Nanjing, China, RC112-01) according to the manufacturer’s instructions. RNA integrity was assessed using an Agilent 2100 Bioanalyzer (Agilent Technologies, Santa Clara, CA, USA), and samples with RNA Integrity Number (RIN) greater than or equal to 7.0 were selected for subsequent analysis.

After RNA quality control, mRNA was enriched from total RNA using HieffNGS™ mRNA Isolation Master Kit (Yeasen, Beijing, China, Cat#12603) with oligo(dT)-conjugated magnetic beads. The enriched mRNA was then fragmented into short pieces using the fragmentation buffer. First-strand cDNA was synthesized using random hexamer primers, followed by second-strand synthesis with dNTPs, RNase H, and DNA Polymerase I. The resulting double-stranded cDNA was subjected to end repair, A-tailing, and ligation of sequencing adapters. After adapter ligation, the cDNA libraries were amplified by PCR and purified. The final libraries were quantified using a Qubit Fluorometer (Thermo Fisher Scientific, Waltham, MA, USA) and assessed for fragment size distribution using an Agilent 2100 Bioanalyzer (Agilent Technologies, Santa Clara, CA, USA). Libraries that passed quality control were used for sequencing. Paired-end sequencing (150 bp) was performed on the DNBSEQ-T7 platform (BGI, Beijing, China). On average, approximately 10.27 GB of clean data were generated per sample.

### 2.13. Statistical Analysis

GraphPad Prism 8.0 software (GraphPad Software, San Diego, CA, USA) and SPSS Statistics 26.0 (IBM Corp., Armonk, NY, USA) software were used for data plotting and statistical analysis, respectively. Data results are expressed as mean plus or minus standard deviation. Statistical comparisons were performed using *t*-tests, and a significance level of *p* < 0.05 was considered statistically significant. For multiple group comparisons (e.g., Figure 2E and Figure 3A), one-way ANOVA was applied, followed by Tukey’s honestly significant difference (HSD) post hoc test for pairwise comparisons. Survival curves were analyzed using the log-rank test. For RNA-seq analysis, differentially expressed genes were identified using DESeq2 (v1.40.2; Bioconductor) with a false discovery rate (FDR) < 0.05 and absolute log2 fold change greater than or equal to 1 as thresholds. A *p* value of less than 0.05 was considered statistically significant.

## 3. Results

### 3.1. Knockout of PIKfyve in Intestinal Epithelial Cells Had No Significant Impact on the Structural Integrity of the Mouse Intestines

By assessing the levels of PIKfyve protein in tissues, we confirmed the specific deletion of PIKfyve only in the intestine (Figure 1A). And the deletion of PIKfyve results in the accumulation of vesicles in the intestinal epithelium, indicative of significant vacuolization pathology (Figure 1B). Subsequently, we evaluated the impact of PIKfyve deletion on mouse intestinal villi and crypts. In PIKfyve cKO mice, the arrangement of intestinal villi was compact, and crypts were intact and in a normal proliferative state, showing no significant alterations compared to the control group, except for vacuolization (Figure 1C–E). These suggest that PIKfyve deletion in intestinal epithelium has no discernible impact on the structure and function of the intestine.

### 3.2. Deletion of PIKfyve in Intestinal Epithelial Cells Exacerbates Post-Irradiation Intestinal Injury and Impedes the Recovery of Intestinal Stem Cells

However, following localized abdominal irradiation at 10 Gy, PIKfyve cKO mice exhibited significant villus sloughing and pronounced contraction, along with severe disruption of intestinal epithelial structure compared to the control group (Figure 2A,B). Notably, in PIKfyve knockout mice, irradiation resulted in compromised crypt morphology and reduced proliferative crypt numbers (Figure 2A,C), potentially disrupting the normal renewal of intestinal epithelial cells. Staining of intestinal stem cells revealed a significant reduction in stem cell markers within the crypts of PIKfyve cKO mice, which could severely impair normal crypt function (Figure 2D,E). These results indicate that PIKfyve deletion leads to structural damage in the intestines of irradiated mice, with impaired timely repair processes. To assess the impact of PIKfyve deletion on post-irradiation survival, a separate cohort of mice was exposed to 13 Gy abdominal irradiation-a dose selected to achieve measurable mortality differences within the 14-day observation window while remaining sub-lethal for wild-type mice, as 10 Gy produced insufficient mortality in controls to discern genotype-specific survival differences. PIKfyve-cKO mice exhibited markedly heightened post-irradiation mortality due to a compromised epithelial barrier and severe mucosal disintegration that facilitates bacterial invasion and nutrient loss (Figure 2F).

### 3.3. Intestinal Epithelial PIKfyve Deletion Compromises Post-Irradiation Barrier Function in Murine Gut

After radiation-induced damage to the intestine, injury to epithelial and endothelial cells leads to barrier dysfunction [13], subsequently triggering a severe inflammatory response in the intestine. Intestinal immune response and inflammation are generally considered crucial mechanisms in the development of radiation-induced intestinal damage.

Through the measurement of FITC-dextran levels in the peripheral blood of mice, we observed that under steady-state conditions, PIKfyve deletion does not affect intestinal permeability, enabling resistance against bacterial invasion and preventing the onset of severe inflammatory reactions (Figure 3A,B). After irradiation, PIKfyve-cKO mice exhibit profound mucosal-barrier disruption and elevated intestinal permeability. To further evaluate the integrity of the intestinal epithelial barrier, we performed immunofluorescence staining for the tight junction-associated protein Claudin-1. At 6 h and 24 h after irradiation, no obvious difference in Claudin-1 expression was observed in the intestinal epithelium between the cKO and WT groups. However, at 3.5 d post-irradiation, Claudin-1 expression in the intestinal epithelium of the cKO group was clearly reduced compared with the WT group, indicating severe damage to the intestinal epithelial barrier (Figure 3B). Consequently, the intestine fails to establish an immune barrier, resulting in severe immune-inflammatory responses (Figure 3C,D). These findings confirm that deletion of PIKfyve in intestinal epithelial cells increases radiation sensitivity in mice.

Radiation exposure increases the apoptosis rate of intestinal epithelial cells and crypt cells, and significant crypt apoptosis typically leads to impaired intestinal epithelial regeneration [14], resulting in compromised function. By assessing changes in apoptosis-related markers at 6 h post-irradiation, we found that under steady-state conditions, there was no significant impact on mouse intestinal crypt cells after PIKfyve deletion compared to the control group. However, following 10 Gy irradiation, there was a significant increase in Cleaved-Caspase3 and TUNEL levels in PIKfyve cKO intestinal crypt cells (Figure 3E–H), indicating that PIKfyve deletion increases apoptosis of intestinal crypt cells post-irradiation. This, to some extent, explains the phenomenon of epithelial dysfunction observed after PIKfyve deletion.

### 3.4. PIKfyve cKO Exacerbates Apoptosis and Inflammatory Responses in Post-Irradiation Murine Enteroids

Following the induction protocol, PIKfyve^fl/fl^;Villin-Cre/ER^T2^ mice received tamoxifen to ablate PIKfyve specifically in the intestinal epithelium. Intestinal crypts were then isolated and expanded as organoids. Passaged organoids were irradiated with 3 Gy on day 1, and growth was monitored. In unirradiated cultures, both genotypes proliferated normally and generated buds (Figure 4A). After irradiation, PIKfyve-deficient organoids displayed reduced expansion and markedly fewer buds, indicating pronounced growth inhibition (Figure 4B,C). To define the role of PIKfyve in murine intestinal radiation injury, we performed RNA sequencing on small-intestinal organoids from PIKfyve-knockout and control mice at 6 and 24 h after irradiation. Differential expression was depicted by volcano and heat-map plots (Figure 4D,E). At 6 h, 347 genes (e.g., Reg1, Reg3b, Reg3g, Cox6b2) were down-regulated and 154 genes (e.g., Parp3, Alox12b, Alox5ap, Mcoln3, Ace) were up-regulated. By 24 h, 401 genes (e.g., Dmbt1, Ctse, Cdh3) were down-regulated, and 152 genes (e.g., Parp3, Rac2, Bmx, Defa2, Defa33) were up-regulated. KEGG enrichment placed Arachidonic acid metabolism and Fc-gamma receptor-mediated phagocytosis among the top pathways at 6 h (Figure 4F), aligning with the pronounced DNA-damage and inflammatory signatures observed in PIKfyve-deficient organoids [15,16]. GSEA enrichment analysis revealed that genes related to inflammatory responses and apoptosis were highly enriched in the irradiated knockout group (Figure 4G). These pathways remained within the top 20 at 24 h, indicating that PIKfyve loss intensifies and prolongs inflammation, thereby delaying epithelial repair and amplifying radiation-induced gut damage. It should be noted that intestinal organoids lack immune cells; therefore, the observed inflammatory signatures likely reflect epithelial-intrinsic stress responses rather than immune cell-mediated inflammation. In summary, intestinal epithelial PIKfyve deletion amplifies radiation enteropathy by sustaining Alox12b- and Rac2-driven inflammation while repressing Reg1-mediated repair, thereby intensifying crypt apoptosis, compromising barrier integrity, and increasing mortality.

To validate the inflammatory signatures identified by RNA-seq in vivo, we performed immunofluorescence staining for Alox12b in intestinal tissues at 6 h and 24 h after irradiation. Alox12b expression was increased in WT mice at 6 h post-irradiation, suggesting the initiation of inflammatory cell infiltration (Figure 4H,I). Moreover, Alox12b expression was higher in the cKO group than in the WT group. At 24 h post-irradiation, Alox12b expression in the cKO group remained persistently elevated, indicating sustained inflammatory cell infiltration (Figure 4H,I).

## 4. Discussion

**Figure 5:**
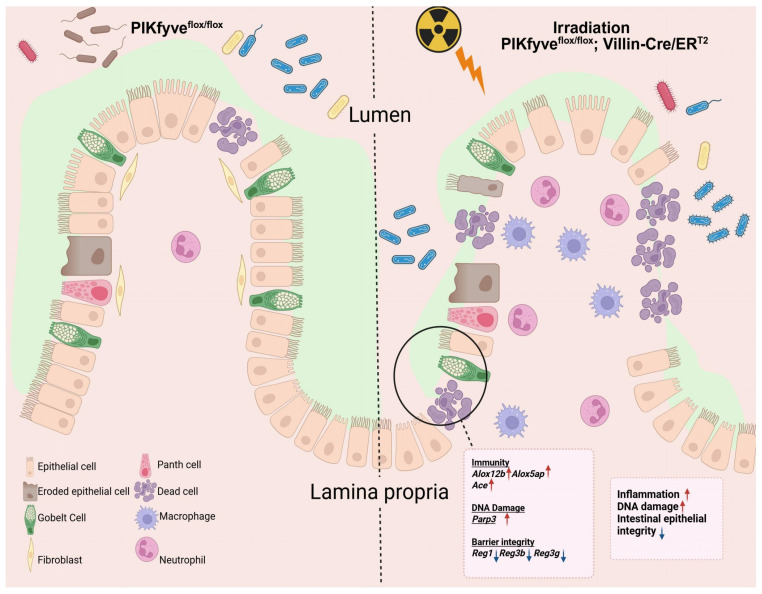
Model elucidating the impact of intestinal epithelial PIKfyve deletion on the radiosensitivity of the murine small intestine. Under homeostatic conditions, intestinal epithelial lineages coordinate to preserve barrier integrity. Ionizing radiation disrupts this coordination, impairing cellular function and increasing permeability. PIKfyve deletion further compromises DNA repair and epithelial restitution, amplifying structural defects. Luminal microbes and microbial products consequently translocate across the mucosa, inciting a pronounced inflammatory response that augments intestinal radiosensitivity. The principal genes involved are illustrated in the figure.

In this study, we identify PIKfyve as a critical guardian of intestinal epithelial integrity against radiation-induced toxicity. Our findings demonstrate that inducible ablation of PIKfyve in intestinal epithelial cells precipitates severe post-irradiation damage characterized by crypt stem cell depletion, exacerbated apoptosis, barrier dysfunction, and lethal inflammation. These results establish PIKfyve-mediated endolysosomal homeostasis as an essential component of the intestinal DNA damage response and position this kinase as a potential target for radiation toxicity mitigation. A schematic summary of these findings is provided in Figure 5.

Previous studies by Takasuga et al. have elucidated essential roles for PIKfyve in mammalian development and adult intestinal homeostasis using germline and constitutive intestine-specific knockout strategies [16]. In their landmark study, systemic PIKfyve deficiency resulted in pre-implantation or early embryonic lethality (by E8.5) due to failure of visceral endoderm nutrient transport, whereas constitutive intestinal epithelial deletion (VilCre-driven) caused spontaneous severe malnutrition, bloody stool, diarrhea, and inflammation resembling Crohn’s disease, with 80% mortality by 100 days of age. While these models elegantly demonstrated PIKfyve’s necessity for embryonic development and basal intestinal function, the profound developmental defects and spontaneous pathology preclude their use as platforms to specifically evaluate radiation sensitivity in adult intestines.

To circumvent these developmental confounders and specifically interrogate PIKfyve’s role in radiation response, we employed a tamoxifen-inducible Cre-LoxP system (Villin-Cre/ER^T2^) to achieve PIKfyve deletion in adult intestinal epithelium prior to irradiation. This strategy allowed us to isolate PIKfyve’s specific function in DNA damage repair and regenerative responses without the compounding effects of developmental abnormalities or pre-existing intestinal inflammation. Notably, under steady-state conditions, our inducible PIKfyve cKO mice exhibited normal intestinal architecture and barrier function despite vacuolization, indicating that acute loss of PIKfyve in mature intestines is tolerated until challenged by radiation stress. This distinction underscores the utility of our inducible model for dissecting radiation-specific vulnerabilities while avoiding the lethal developmental phenotypes observed in constitutive knockouts.

The intestinal epithelium’s remarkable regenerative capacity depends on Lgr5^+^ crypt base columnar stem cells, which drive continuous epithelial renewal. Olfm4 (Olfactomedin-4) has emerged as a robust and specific marker for these intestinal stem cells, serving as a critical readout for crypt regenerative capacity following genotoxic injury [14,17]. In our study, radiation exposure in PIKfyve-deficient intestines resulted in a dramatic reduction of Olfm4^+^ stem cells compared to controls, indicating that PIKfyve is indispensable for maintaining the stem cell pool under radiation stress. This depletion of Olfm4-positive cells likely represents the primary driver of the observed crypt hypoplasia and impaired epithelial restitution, as the loss of these regenerative progenitors compromises the intestine’s ability to replace damaged epithelium, ultimately leading to barrier collapse and lethal sepsis.

Our findings indicate that post-irradiation PIKfyve deletion exacerbates inflammation, perpetuating intestinal damage and exacerbating post-irradiation mortality in mice. These effects may be attributed to increased apoptosis of intestinal crypt cells in PIKfyve knockout mice following irradiation. To dissect the role of PIKfyve in radiation-induced intestinal injury, we derived small-intestinal organoids from PIKfyve-cKO mice. Irradiation markedly impaired their growth. Transcriptomic profiling of organoids harvested at sequential intervals after exposure revealed persistent up-regulation of the DNA-repair gene Parp3, indicative of ongoing DNA damage. Concurrently, Alox12b and Alox5ap—encoding enzymes that generate leukotrienes and lipoxins—were elevated, signifying an acute inflammatory state. Enhanced angiotensin-converting enzyme (Ace) expression further promoted vasoconstriction and increased mucosal permeability, amplifying the inflammatory cascade and, consequently, radiosensitivity. Notably, the regenerative protein Reg1 was down-regulated in PIKfyve-deficient organoids and was almost abolished 24 h after irradiation. Reg1, induced by IL-6/IL-22 via STAT3, stimulates crypt-cell proliferation and fortifies the epithelial barrier by up-regulating claudin-3/4, thereby limiting bacterial translocation. Loss of PIKfyve blunted this reparative axis, resulting in defective epithelial renewal, heightened permeability, and sustained post-irradiation inflammation that exacerbated intestinal damage.

While our transcriptomic data demonstrate potentiated DNA-damage signaling in PIKfyve-deficient organoids, the mechanistic link between PIKfyve loss and enhanced radiation-induced DNA damage remains correlative rather than causative. PIKfyve-generated PI(3,5)P2 is essential for late endosome and lysosome maturation, and its deficiency impairs autophagic flux and lysosomal degradation [18]. Autophagy plays a critical role in maintaining genomic integrity by clearing damaged mitochondria, thereby reducing reactive oxygen species that exacerbate DNA lesions, and by regulating the turnover of DNA repair proteins [19]. We hypothesize that PIKfyve deficiency disrupts autophagy-dependent clearance of radiation-induced cellular damage, leading to accumulation of oxidative stress and impairment of homologous recombination repair, which is particularly important for resolving replication-associated double-strand breaks in rapidly proliferating crypt cells [20]. This interpretation is consistent with the observed up-regulation of Parp3 in our RNA-seq data, which likely reflects persistent, unresolved DNA damage rather than active repair. Nevertheless, direct evidence, such as gamma-H2AX kinetics, ATM/ATR activation status, or comet assay, will be required to firmly establish whether PIKfyve directly modulates the DNA damage response machinery or whether the enhanced DNA-damage signaling is secondary to lysosomal dysfunction and autophagy arrest.

The relative contribution of increased crypt apoptosis versus stem cell loss to the observed phenotype warrants careful consideration. In our model, cleaved-caspase-3 staining was markedly elevated as early as 6 h post-irradiation, whereas Claudin-1 immunofluorescence remained unchanged at 6 h and 24 h, with clear reduction only evident by 3.5 d. This temporal pattern suggests that acute crypt apoptosis precedes overt barrier breakdown, arguing that PIKfyve deficiency may lower the apoptotic threshold of crypt epithelial cells under radiation stress. Crypt base columnar stem cells, marked by Olfm4, are exquisitely sensitive to radiation-induced apoptosis, yet they also possess remarkable regenerative plasticity; surviving stem cells or their progeny can repopulate damaged crypts through dedifferentiation [17,21]. The dramatic reduction of Olfm4+ cells in PIKfyve-cKO mice may therefore reflect either primary stem cell death due to defective DNA repair or, alternatively, a secondary consequence of widespread apoptosis and inflammatory destruction of the stem cell niche. Distinguishing between these possibilities will require lineage-tracing experiments or stem cell-focused survival assays to determine whether PIKfyve loss primarily compromises stem cell viability or instead creates a hostile microenvironment that prevents stem cell regeneration.

The temporal relationship between barrier dysfunction and inflammation in our model appears more complex than a simple unidirectional cascade. The observation that Alox12b-a marker of inflammatory cell infiltration-was already elevated at 6 h post-irradiation, while Claudin-1 expression remained intact until 3.5 d, challenges the assumption that barrier breakdown is the initiating event. Instead, these data suggest that PIKfyve deficiency may amplify early inflammatory signaling, which subsequently compromises tight junction integrity. This interpretation is biologically plausible given that PIKfyve-generated PI(3,5)P2 regulates endosomal maturation and trafficking of Toll-like receptors, which are critical sensors of microbial components and damage-associated molecular patterns [22]. Impaired PIKfyve function could alter TLR trafficking and signaling, leading to exaggerated NF-kappaB-driven cytokine production and subsequent tight junction disruption, as pro-inflammatory cytokines such as TNF-alpha and IFN-gamma are known to induce internalization of junctional proteins, including occludin and claudins [23]. Thus, rather than barrier failure triggering inflammation, our data support a model in which PIKfyve deficiency licenses early, epithelial-intrinsic inflammatory signaling that progressively erodes barrier function and creates a self-amplifying cycle of damage.

The profound vacuolization observed in PIKfyve-deficient intestinal epithelium under homeostatic conditions underscores the central importance of this kinase in endolysosomal homeostasis. PI(3,5)P2, the lipid product of PIKfyve, is required for proper vacuole/lysosome membrane fission, acidification, and retrograde trafficking from endosomes to the trans-Golgi network [24]. Consequently, PIKfyve loss leads to characteristic swollen endolysosomal compartments that impair autophagosome-lysosome fusion and block autophagic degradation [25]. Under radiation stress, intact autophagy is essential for clearing damaged organelles and preventing the accumulation of reactive oxygen species that would otherwise exacerbate DNA damage and trigger apoptotic cell death. The absence of PIKfyve may therefore create a “double jeopardy” scenario: impaired lysosomal function not only prevents efficient recycling of cellular components but also sensitizes cells to radiation by compromising the autophagy-dependent cytoprotective response. While our study did not directly assess autophagic flux or lysosome acidification, the established literature strongly supports the hypothesis that PIKfyve-mediated endolysosomal function represents a critical, yet underexplored, determinant of intestinal radio-resistance.

Several critical questions arising from our findings merit investigation in future studies. First, direct assessment of DNA damage repair kinetics, including gamma-H2AX foci resolution, ATM/ATR phosphorylation status, and comet assay, will be essential to determine whether PIKfyve loss directly compromises the DNA damage response or merely amplifies damage secondary to lysosomal dysfunction. Second, lineage-tracing experiments using Lgr5-CreERT2 or Olfm4-CreERT2 mice combined with caspase inhibition could dissect whether stem cell depletion is a primary consequence of PIKfyve deletion or a secondary result of niche destruction by widespread apoptosis. Third, a comprehensive time-course analysis incorporating FD4 permeability, cytokine profiling, and tight junction protein immunostaining at 6 h, 12 h, and 24 h post-irradiation will clarify the temporal sequence of barrier breakdown and inflammation. Fourth, direct assessment of autophagic flux, such as LC3 turnover assays, p62 accumulation, and lysosomal pH measurements, would test the hypothesis that defective autophagy underlies the radiosensitization observed in PIKfyve-deficient intestines. Addressing these questions will not only strengthen the mechanistic framework of our findings but may also reveal novel therapeutic targets for mitigating radiation-induced gastrointestinal toxicity.

The translational significance of our findings is underscored by the emerging clinical development of PIKfyve inhibitors as cancer therapeutics. Apilimod, a first-in-class PIKfyve kinase inhibitor, has demonstrated promising efficacy in Phase I/II clinical trials for B-cell non-Hodgkin lymphoma, Crohn’s disease, and amyotrophic lateral sclerosis [26,27,28]. Additionally, preclinical studies have revealed that PIKfyve inhibition can enhance antitumor immunity by upregulating MHC-I expression and potentiating immune checkpoint blockade in prostate cancer models [29,30]. Other PIKfyve-targeting agents currently under investigation include next-generation inhibitors with improved pharmacokinetic properties.

Given the concurrent use of radiotherapy in the management of these malignancies, our results raise critical safety concerns regarding the potential for PIKfyve inhibitors to exacerbate radiation-induced gastrointestinal toxicity. The heightened radiosensitivity observed in our inducible knockout model suggests that patients receiving PIKfyve inhibitors may experience enhanced intestinal toxicity, including severe diarrhea, mucositis, and potentially life-threatening enteritis, when undergoing abdominal or pelvic radiotherapy. This interplay necessitates careful optimization of dosing schedules to temporally separate PIKfyve inhibition from radiation exposure, or the implementation of prophylactic intestinal radioprotective strategies in combined modality protocols. Conversely, our findings also suggest that transient, localized PIKfyve inhibition might sensitize intestinal tumors to radiation while sparing normal tissue, though this therapeutic window requires careful preclinical validation.

## 5. Conclusions

In summary, our study establishes PIKfyve as a previously unrecognized protective factor against radiation-induced intestinal toxicity. By utilizing an inducible knockout strategy that avoids the developmental lethality of germline deletion, we demonstrate that PIKfyve maintains crypt stem cell viability, limits apoptotic cell death, and preserves barrier integrity following radiation exposure. These findings not only advance our understanding of intestinal radiobiology but also provide crucial guidance for the clinical development of PIKfyve-targeted therapies, emphasizing the need for rigorous assessment of radiation toxicity risks in patients undergoing combined treatment regimens.

## Figures and Tables

**Table 2:** The sequences of primers for RT-PCR.

Gene	Primer	Sequence (5′→3′)
TNF-α	F	CAGGCGGTGCCTATGTCTC
	R	CGATCACCCCGAAGTTCAGTAG
TGF-β	F	GGCCAGATCCTGTCCAAGC
	R	GTGGGTTTCCACCATTAGCAC
IL-6	F	CTGCAAGAGACTTCCATCCAG
	R	AGTGGTATAGACAGGTCTGTTGG
CXCL5	F	GTTCCATCTCGCCATTCATGC
	R	GCGGCTATGACTGAGGAAGG
CCL2	F	TAAAAACCTGGATCGGAACCAAA
	R	GCATTAGCTTCAGATTTACGGGT
IL-1β	F	ATGATGGCTTATTACAGTGGCAA
	R	GTCGGAGATTCGTAGCTGGA
GAPDH	F	ATGGTGAAGGTCGGTGTGAA
	R	TGGAAGATGGTGATGGGCTT

**Figure A1:**
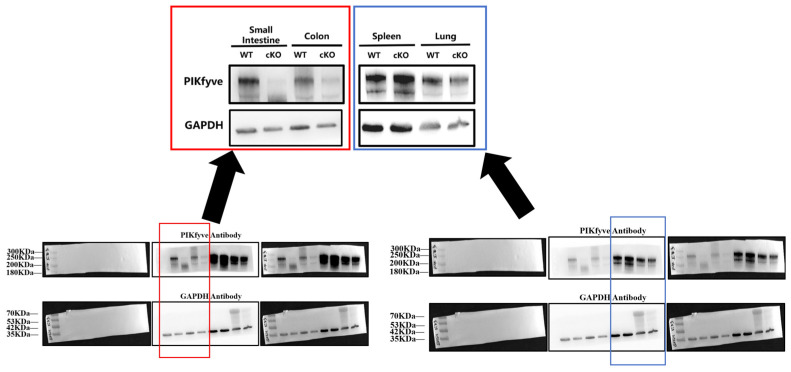
Western blot original data for Figure 1A. Full-length blots showing PIKfyve and GAPDH bands from intestine, colon, spleen, and lung tissues of PIKfyve^fl/fl^ and PIKfyve^fl/fl^;Villin-Cre/ER^T2^ mice.

## Data Availability

The datasets used and/or analyzed during the current study are available from the corresponding author on reasonable request. The raw RNA-seq data reported in this study have been deposited in the NCBI Sequence Read Archive (SRA) under BioProject accession number PRJNA1428555. Processed RNA-seq data, including normalized count tables, differential expression results with FDR-corrected *p* values, and complete KEGG/GSEA enrichment outputs, are provided in Supplementary Data Files S1–S4. The data that support the findings of this study are openly available in Zenodo at https://doi.org/10.5281/zenodo.20039074.

## Supplementary Materials

The following supporting information can be downloaded at: https://www.mdpi.com/article/10.3390/toxics14050434/s1, File S1: Normalized_Counts; File S2: DE_Results; File S3: KEGG_Enrichment; File S4: GSEA_Results.

## Abbreviations

The following abbreviations are used in this manuscript:

Ace	angiotensin-converting enzyme
Alox12b	arachidonate lipoxygenase 12B
Alox5ap	arachidonate 5-lipoxygenase-activating protein
BrdU	bromodeoxyuridine
CCL2	C-C motif chemokine ligand 2
cKO	conditional knockout
CreERT2	Cre-estrogen receptor T2
CXCL5	C-X-C motif chemokine ligand 5
DAB	3,3′-diaminobenzidine
EDTA	ethylenediaminetetraacetic acid
FD4	fluorescein isothiocyanate-dextran (4 kDa)
Fig	figure
GAPDH	glyceraldehyde-3-phosphate dehydrogenase
GSEA	Gene Set Enrichment Analysis
Gy	Gray
HE	hematoxylin and eosin
IARS	intestinal acute radiation syndrome
IL	interleukin
KEGG	Kyoto Encyclopedia of Genes and Genomes
Lgr5	leucine-rich repeat-containing G-protein coupled receptor 5
MHC-I	major histocompatibility complex class I
NC	nitrocellulose
ns	not significant
Olfm4	olfactomedin-4
PARP3	poly(ADP-ribose) polymerase 3
PBS	phosphate-buffered saline
PI(3,5)P2	phosphatidylinositol-3,5-bisphosphate
PI3P	phosphatidylinositol-3-phosphate
PIKfyve	phosphoinositide kinase, FYVE-type zinc finger containing
qPCR	quantitative polymerase chain reaction
Reg	regenerating gene
RIN	RNA integrity number
RT-qPCR	reverse transcription quantitative PCR
SPF	specific pathogen-free
STAT3	signal transducer and activator of transcription 3
TBST	Tris-buffered saline with Tween-20
TGF-β	transforming growth factor-beta
TNF-α	tumor necrosis factor-alpha
TUNEL	terminal deoxynucleotidyl transferase dUTP nick end labeling
WT	wild type
μL	microliter
μm	micrometer
